# Rating scales to measure adverse effects of medications in people with intellectual disability: a scoping review

**DOI:** 10.1007/s00228-022-03375-2

**Published:** 2022-08-31

**Authors:** Neasa Kelly, Andrew Kilmartin, Kevin Lannon, Caren Lee, Rory McLoughlin, Lara Mulvanny, Omnyiah Mohamed, Mairead Treacy, Karen Rossi, Juliette O’Connell

**Affiliations:** School of Pharmacy & Pharmaceutical Sciences, Trinity College, Dublin, Ireland

**Keywords:** Intellectual disability, Rating scales, Adverse medication effects, Medication

## Abstract

**Purpose:**

Intellectual disability (ID) is a chronic neurodevelopmental condition characterised by limitations in intelligence and adaptive skills with an onset prior to the age of 18 years. People with ID have complex healthcare needs and are more likely than the general population to experience multiple comorbidities and polypharmacy, with subsequent increased risk of adverse medication effects. The aim of this scoping review is to characterise rating scales used to measure adverse effects of medication in people with ID.

**Methods:**

Four online databases (PsycINFO, Medline, Web of Science and OpenGrey) were searched in April 2020. Studies were assessed for inclusion against pre-specified eligibility criteria. Reference lists of included studies were hand searched. Data extraction was carried out by two independent reviewers and key findings were tabulated for consideration. Studies were assessed for quality using the Mixed Methods Appraisal Tool.

**Results:**

The search resulted in 512 unique records, of which fifteen met the inclusion criteria. Fourteen scales were identified. All scales assessed adverse effects of psychotropics only. Of the scales, only one, the Matson Evaluation of Drug Side Effects, which focuses on psychotropic medications, was originally developed for use in a population with ID.

**Conclusion:**

The Matson Evaluation of Drug Side Effects scale appears to be the most reliable and well-researched scale in people with ID. However, a scale which measures adverse effects across multiple medication classes would be valuable for use in this population.

**Supplementary Information:**

The online version contains supplementary material available at 10.1007/s00228-022-03375-2.

## Introduction

Intellectual disability (ID) is a chronic neurodevelopmental condition characterised by limitations in intelligence and adaptive skills with an onset prior to the age of 18 years [1].

People with ID have complex healthcare needs and are 2.5 times more likely to experience multiple health problems [2, 3]. A cross-sectional study of 753 people with ID over age 40 found that 71.2% of participants lived with multiple morbidities. The most prevalent of these include mental illness, gastrointestinal disorders, neurological disorders and ocular disease [3]. People with ID are particularly susceptible to polypharmacy due to the extensive medication regimens needed to manage these conditions [4]. Subsequently, people with ID have a high medication burden, especially from anticholinergics, sedatives and psychotropic medications, including antipsychotics [5–11]. While first-generation antipsychotics are known to cause a range of extrapyramidal symptoms, such as akathisia, tardive dyskinesia (TD) and dystonia, newer generation atypical antipsychotics produce cardiovascular and metabolic adverse effects [12]. The prevalence of epilepsy in people with ID is approximately 22%, compared to 1.1% in the general population [13–15]. Antiepileptic medications are often used by people with ID due to the higher prevalence of epilepsy and for their mood stabilisation effects. However, these medications can cause sedation and drowsiness in addition to producing anticholinergic effects [16]. People who have ID are also more likely to experience drug-related adverse effects [17].

It is not appropriate to extrapolate data on adverse effects of medications for people with ID from the general population. This is due to confounding factors such as existing cognitive disabilities and communication difficulties [18], in addition to evidence suggesting that people with ID may have different susceptibility to medication adverse effects. People with ID have been found to be more likely to suffer from movement related adverse effects from antipsychotics in recent UK evidence [19]. Drug-drug interactions, increased body fat, neurological damage, genetic abnormalities and differences in expression of metabolic enzymes responsible for drug degradation are factors which may contribute to altered effects, and adverse effects, of medications in this population [20–22]. These adverse effects can have serious clinical consequences, including hospitalisation [23]. Polypharmacy has also been found to be a predictor for mortality in older adults with ID [24].

There are further unique challenges in the care of people with ID. Verbal and non-verbal individuals struggle with communication from childhood into older age, and healthcare professionals may lack knowledge and skills to effectively interact with people with ID [25–27]. This has led to a disparity in the efficacy of receptive and expressive communication between people with ID and healthcare providers [28]. This causes difficulties in obtaining accurate information during medication use reviews and the detection of adverse drug effects, especially for symptoms that are more difficult to identify, for example neurological damage [17, 29]. Failure to detect adverse drug events can contribute to worsening quality of life [30]. Diminished cognitive and language skills make it difficult to interpret subjective symptoms of adverse medication effects and objective symptoms may be misattributed to other health conditions, leading to diagnostic overshadowing and incremental prescribing [31–33]. As a result, assessment of adverse medication effects often relies on observable behaviours, which may be direct (involving clinical assessment of the person by a trained healthcare professional), or indirect, by interview with a carer [32].

As a potential solution to these difficulties, adverse effect rating scales for medicines frequently used by people with ID have been investigated in this population. These include scales developed specifically for use in people with ID (e.g. the Matson Evaluation of Drug Side Effects (MEDS) [17, 34–40]) or modified versions of scales developed for the general population (e.g. the Udvalg for Kliniske Undersøgelser (UKU) Rating Scale [41]). These scales aim to objectively identify cognitive and physical adverse medication effects and determine their severity. While scales alone cannot be used to yield a diagnosis of the adverse medication effect, they can be valuable as a screening tool to improve medication monitoring in a population which can be hindered by difficulties in communication [32, 36].

To date, no clear review of the characteristics or psychometric properties of scales used to assess adverse medication effects in people with ID has been conducted.

This scoping review of the literature aims to characterise the existing rating scales used to measure the adverse effects of medication in people living with ID and assess the best approach for their use in practice.

The objectives of the study are as follows:

1. To determine the adverse medication effect scales available for use in people with ID
2. To review the medication classes included in these scales
3. To identify the types of adverse medication effects recognised in each of the scales; and
4. To explore the robustness of the scales in terms of their psychometric properties, reliability, and validity.

## Method

### Search strategy

A search of studies which used rating scales to determine adverse effects of medication in people with ID was conducted in accordance with the “Preferred Reporting Items for Systematic Reviews and Meta-Analyses” extension for scoping reviews (PRISMA-ScR) guidelines [42].

The electronic literature databases selected for this review were PsycINFO, Medline, Web of Science and OpenGrey. These databases were searched on 4th and 5th April 2020.

Relevant search terms (keywords) and controlled vocabulary related to the core concepts of the research question were developed with reference to relevant literature [43, 44]. The core concepts were (1) people with ID, (2) medication adverse effects and (3) measures or specific rating scales. The search strategy and specific parameters are summarized in Online Resource 1 of the Electronic Supplementary Material.

Hand searching of bibliographies was also performed on articles selected for inclusion. Forward and backward searches were completed by two independent reviewers in April 2020.

### Screening and eligibility

Titles and abstracts were screened for relevance by two reviewers and conflicts were resolved by a third independent party. Selection for more in-depth screening was determined by the inclusion and exclusion criteria outlined in Table 1.

**Table 1:** Summary of inclusion and exclusion criteria applied to retrieved articles

**Inclusion criteria**	**Exclusion criteria**
(a) Studies published in English	For title/abstract: (a) animal studies or non-human studies (b) studies which include participants other than people with intellectual disability (c) articles in which no rating scale to measure medication adverse effects are utilised (d) articles not exploring adverse effects or side effects (e) An adverse effect that was not measurable with a questionnaire or rating scale
(b) The study participants have intellectual disability according to: (i) The Diagnostic and Statistical Manual of Mental Disorders, fourth or fifth revision (DSM-IV, DSM-V) (ii) The International Statistical Classification of Diseases and Related Health Problems, 10^th^ or 11^th^ revision (ICD-10, ICD-11)	
(c) Reported adverse effects as primary or secondary outcomes of medication use	
(d) Studies utilising a control or reference group as a comparator	
(e) A rating scale must be employed in the study to assess the medication-related adverse effect	
(f) Studies eligible for inclusion: (i) Peer reviewed, published articles (ii) Grey literature	Additional exclusion criteria selected for full-text review: (f) article not about an adverse effect scale in people with intellectual disability (g) full text not available (h) language other than English
(g) Published data is to be limited to those produced within the last 20-year period	Data sources excluded from the review: (i) Abstract only/full text not available (ii) Review articles (iii) Editorials (iv) Letters (v) Conference papers

Articles retained from title and abstract screening proceeded to full text screening. Full text screening was performed by two reviewers and disagreements were resolved by a third independent party. Eligibility was determined by the previously described inclusion and exclusion criteria. Data were extracted from the selected studies by two further reviewers and relevant details were tabulated into a concise format for consideration.

### Quality assessment

The Mixed Methods Appraisal Tool (MMAT) was used to assess the quality of the studies included in this scoping review [45]. Two reviewers undertook the quality assessment of the studies chosen for inclusion in this scoping review.

## Results

### Search results

**Fig. 1:**
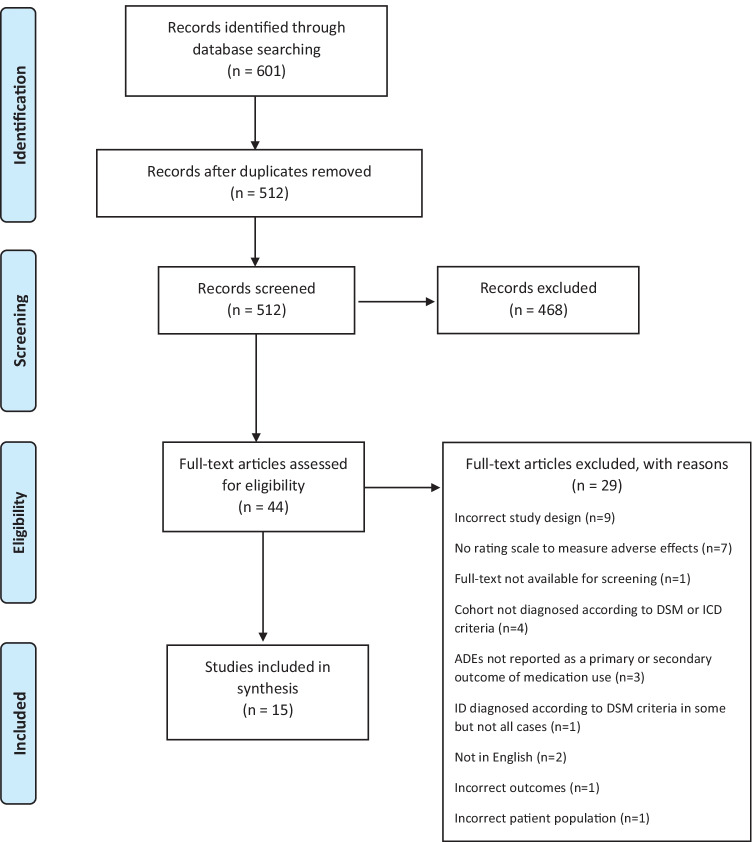
PRISMA flow diagram

After screening 512 titles and abstracts and 44 full text documents, 15 unique studies across 16 publications were included in the final review, with each article meeting the predetermined inclusion criteria. Figure 1 presents a PRISMA flow diagram of the results.

### Characteristics of included studies

All studies evaluated utilised a rating scale to measure adverse effects of medication in a population with ID. All studies were published in the last 20 years and the majority originated in the United States (US) (*n* = 11), with 8 of these based in the same developmental centres in the South-Eastern US (Louisiana). A choropleth of study location by geographic region is included in Online Resource 2*.* Most studies were observational in nature (*n* = 12), while three studies were clinical trials. A brief description of each scale included in the studies discussed is provided in Online Resource 3. Design characteristics of included studies are detailed in Table 2.

**Table 2:** Summary of included articles

**Study**	**Study Aim**	**Study design**	**Key sample features**	**Rating Scale(s) used**	**Drug class(es) analysed**	**Key results**
**Brandt et al. **[46]	Assessment of the effects of topiramate on cognitive functioning in patients with epilepsy and ID	Observational cross-sectional study	**Setting:** Tertiary referral centre for epilepsy and ID **Participants:** *n* = 26 **Level of ID:** Mild (*n* = 17; 65%); moderate-severe (*n* = 9; 35%) **Age:** 19 to 67 years **Gender:** 42% male, 58% female	1. RBMT 2. Digit span forward and backward test from HAWIE- R 3. RWT 4. Five-point test 5. Trail making test ‘switching’ condition from D-KEFS 6. Digit symbol test from HAWIE-R	Antiepileptic drug (topiramate)	Patients not treated with topiramate had significantly better cognitive speed, verbal-short term memory, working memory and semantic verbal fluency than those treated with topiramate
**Correia Filho et al. **[47]	To evaluate the short-term efficacy and tolerability of risperidone and methylphenidate for reducing attention deficit hyperactivity disorder (ADHD) related symptoms in children and adolescents with moderate ID	Randomised parallel group clinical trial	**Setting:** Outpatient clinic **Participants:** *n* = 45 **Level of ID:** Moderate **Age:** 6 to 16 years **Gender**: 76% male, 24% female	1. Barkley’s SERS 2. UKU	Antipsychotic (risperidone) and psychostimulant (methylphenidate)	In the methylphenidate group, the SERS total score showed no significant difference between baseline and endpoint In the risperidone group, no statistically significant difference was detected between baseline and endpoint scores for any of the UKU subscale scores. A trend for an increase in neurological effects (extrapyramidal effects) was recognised
**Fodstad et al. **[48]	To compare MEDS Central Nervous System- Parkinsonism/Dyskinesia subscale scores in individuals with ID and Axis I diagnosis to those with ID only	Observational cross-sectional study	**Setting:** Two developmental centres in the South-Eastern US (Louisiana) **Participants:** *n* = 60 **Level of ID:** Mild (6.7%); moderate (13.3%); severe (30%); profound (50%) **Age:** 17 to 80 years **Gender:** 53% male, 47% female	MEDS Central Nervous System- Parkinsonism/Dyskinesia subscale	Antipsychotics (atypical)	Individuals with ID actively taking atypical antipsychotics appear to be at an increased risk of developing symptoms of tardive dyskinesia, regardless of mental illness status
**Garcia **[32] **Garcia et al. **[49]	To evaluate the psychometric properties of the MEDS and ARMS and to further analyse the symptom profile of akathisia in adults with ID	Observational cross-sectional study	**Setting:** Two developmental centres in the South-Eastern US (Louisiana) **Participants:** *n* = 66 **Level of ID:** Moderate (*n* = 5; 8%); severe (*n* = 10; 15%); profound (*n* = 51; 77%) **Age:** 29 to 79 years **Gender:** 45.5% male, 54.5% female	1. MEDS Central Nervous System- Behavioural/Akathisia subscale 2. ARMS	Antipsychotics	The MEDS Central Nervous System- Behavioural/Akathisia subscale and ARMS total score were reliable in establishing which participants evidenced akathisia
**Ghuman et al. **[50]	To assess the safety and efficacy of methylphenidate in children with pervasive developmental disorders and hyperactivity	Crossover randomised controlled trial	**Setting:** Outpatient clinic **Participants:** *n* = 14 **Level of ID:** Not reported **Age:** 3 to 5.9 years **Gender:** 93% male, 7% female	1. SERS 2. Stereotyped Behaviour Scale 3. Yale Global Tick Severity Scale 4. Yale Brown obsessive compulsive scale	Psychostimulant (methylphenidate)	Half of participants experienced side effects, including reports of increased stereotypic behaviour, upset stomach, sleep-related difficulties, and emotional lability
**Hellings et al.** [51]	To investigate the benefit of risperidone for severe disruptive behaviour in patients with autism spectrum disorders and ID	Crossover randomised controlled trial	**Setting:** Outpatient clinic **Participants:** *n* = 40 **Level of ID:** Mild (*n* = 11; 27.5%), moderate (*n* = 9; 22.5%), severe (*n* = 11; 27.5%), profound (*n* = 9; 22.5%) **Age:** 8 to 56 years **Gender:** 57.5% male, 42.5% female	1. NSEC 2. DISCUS	Antipsychotic (risperidone)	The mean DISCUS score did not change significantly throughout the trial
**Hellings et al. **[52]	To examine long-term adverse events of risperidone in children and adults with pervasive developmental disorders and ID	Prospective follow-up from crossover randomised controlled trial	**Setting:** Outpatient clinic **Participants:** *n* = 19 **Level of ID**: Mild (*n* = 5; 26.3%); moderate (*n* = 3; 15.8%); severe (*n* = 4; 21.1%), profound (*n* = 6; 31.6%), severe/profound (*n* = 1; 5.3%) **Age**: 8 to 56 years **Gender:** 57.9% male, 42.1% female	1. NSEC 2. DISCUS	Antipsychotic (risperidone)	Increases in DISCUS and NSEC score were low in the period after week 47
**Hess et al. **[53]	To examine the adverse effects of psychotropic medication in an adult population with ID and autism spectrum disorder	Observational cross-sectional study	**Setting:** Two developmental centres in the South-Eastern US (Louisiana) **Participants**: *n* = 48 **Level of ID:** Severe (2%); profound (98%) **Age:** 28 to 78 years **Gender:** 62% male, 38% female	MEDS	Antiepileptic drugs/mood stabilisers, anxiolytics and atypical antipsychotics	Participants on more psychotropic medications across multiple classes had more adverse effects in comparison to individuals receiving fewer medication classes
**Mahan et al. **[54]	To examine whether number of psychotropic medications, across different psychotropic medication classes, influences adverse effects presentation among adults with ID	Observational cross-sectional study	**Setting:** Inpatient adults at two supports and services centres in the South-Eastern US (Louisiana) **Participants:** *n* = 80 **Level of ID:** Mild (9.8%); moderate (5.95%); severe (13.45%); profound (70.8%) **Age:** 18 to 70 years **Gender:** 55% male, 45% female	MEDS	Antipsychotics (typical and atypical), antiepileptic drugs/mood stabilisers, antidepressants, anxiolytics and antihypertensives	MEDS score analysis revealed that the group taking two psychotropics across multiple medication classes endorsed the most adverse effects
**Matson et al. **[55]	To compare DISCUS scores of persons with ID and an Axis I diagnosis and persons diagnosed with ID without another psychiatric diagnosis	Observational cross-sectional study	**Setting:** Large developmental centre in the South-Eastern US (Louisiana) **Participants:** *n* = 30 **Level of ID:** No Diagnosis: Severe (36.4%); profound (63.6%); Dual Diagnosis: Severe (13.3%); profound (86.7%) **Age:** 33 to 81 years **Gender:** 40% male, 60% female	DISCUS	Antipsychotics, anxiolytics, antidepressants, antiepileptic drugs, beta-blockers, anticholinergics	Individuals with and without psychiatric diagnoses did not differ in total number of dyskinetic symptoms
**Matson et al. **[36]	To examine medication adverse effects exhibited by individuals with ID and a seizure disorder who were actively taking antiepileptic drugs and compare these individuals’ adverse effect profiles with those of a control group	Observational cross-sectional study	**Setting:** Large developmental centre in central Louisiana **Participants:** *n* = 248 **Level of ID:** mild (2.5%), moderate (5%); 18.2% severe (18.2%); profound (68.6%); unspecified (5.5%) **Age:** Mean 52.5 years **Gender:** 42.1% male, 57.9% female	MEDS	Antiepileptic drugs (including phenobarbital, divalproex, carbamazepine, phenytoin, and gabapentin)	Significant differences were observed between the two groups on the Central Nervous System-General and Endocrine and Genitourinary MEDS subscales only, with the medication group exhibiting more endorsement of adverse effects
**Matson et al. ** [39]	To evaluate adverse effects of persons with tardive dyskinesia versus tardive dyskinesia/akathisia	Observational cross-sectional study	**Setting:** Developmental centres in the South-Eastern US (Louisiana) **Participants:** *n* = 161 **Level of ID:** Mild (*n* = 9; 6%); moderate (*n* = 22; 14%); severe (*n* = 21; 13%); profound (*n* = 102; 63%); unspecified (*n* = 7; 4%) **Age**: 18 to 90 years **Gender:** 53% male, 47% female	MEDS	Antipsychotics (atypical) antiepileptic drugs/mood stabilisers, anxiolytics, antidepressants, antihypertensives and antiparkinsonism agents	Profile analysis for the nine MEDS severity subscales revealed significant differences between diagnostic groups. Participants with a diagnosis of tardive dyskinesia/akathisia had the highest adverse effects, followed by those with a tardive dyskinesia diagnosis
**Matson et al. **[38]	To use a psychotropic adverse effect scale to examine dose of Selective Serotonin Reuptake Inhibitors and the effects of adding additional psychotropics in combination with Selective Serotonin Reuptake Inhibitors	Observational cross-sectional study	**Setting:** Two developmental centres in the South-Eastern US (Louisiana) **Participants:** *n* = 49 **Level of ID:** Mild (*n* = 4; 8%); moderate (*n* = 6; 12%); severe (*n* = 5; 10%); profound (*n* = 31; 63%); unspecified (*n* = 3; 6%) **Age:** 18 to 90 years **Gender:** 51% male, 49% female	MEDS	Antidepressants (Selective Serotonin Reuptake Inhibitors)	Individuals using higher doses of Selective Serotonin Reuptake Inhibitors and Selective Serotonin Reuptake Inhibitors plus other psychotropic group had the highest number of adverse effects
**Matson et al. **[40]	Examine adverse effects of atypical antipsychotics over time and adjustments in atypical antipsychotics in people with ID, tardive dyskinesia and akathisia	Prospective observational study	**Setting:** Developmental centre in the South-Eastern US (Louisiana) **Participants:** *n* = 84 **Level of ID:** Mild (*n* = 4; 5%); moderate (*n* = 4; 5%); severe (*n* = 7; 8%); profound (*n* = 62; 74%); unspecified (*n* = 3; 4%) **Age:** 19 to 81 years **Gender:** 60% male, 40% female	MEDS	Antipsychotics (atypical)	Participants with tardive dyskinesia or akathisia had significantly higher adverse effects than participants without these diagnoses
**Tveter et al. **[41]	To utilise a pilot study to investigate whether the UKU-Side Effect Rating Scale (UKU-SERS) is appropriate for use in ID populations	Observational cross-sectional study	**Setting:** Specialist psychiatric in-patient unit for adults with ID **Participants:** *n* = 13 **Level of ID:** Mild (*n* = 9; 69.2%); moderate (*n* = 3; 23.1%); unspecified (*n* = 1; 7.7%) **Age:** 21 to 46 years **Gender:** 61.5% male, 38.5% female 31 years with a range from 21 In total	UKU	Antipsychotics, mood stabilisers and antidepressants	A revised UKU-SERS comprising 35 items could be applicable for patients with ID

*ARMS* Akathisia Ratings of Movement Scale, *DISCUS* Dyskinesia Identification System Condensed User Scale, *D*-*KES* Delis Kaplan Executive Function System, *HAWIE*-*R* Hamburg Wechsler Intelligenztest für Erwachsene, *MEDS* Matson Evaluation of Drug Side-effects, *NSEC* Neurological Side Effect Scale, *SERS* Barkley’s Side Effects Rating Scale, *RBMT* Rivermead Behavioural Memory Test, *RWT* Regensburger Wortflüssigkeitstest; UKU, Udvalg for Kliniske Undersøgelser

### Adverse medication effect scales

Fourteen different adverse effect rating scales were identified which measure both physical and cognitive adverse medication effects. Four scales were examined exclusively in children with ID, seven scales were examined exclusively in adults aged 18 years and over, while three scales have been investigated across both populations (Online Resource 4).

### Medication classes

The medication classes examined across the studies were predominantly nervous system agents: antipsychotics (11 studies), antiepileptic drugs (7 studies), antidepressants (5 studies), anxiolytics (4 studies), antihypertensives (3 studies), psychostimulants (2 studies) and anticholinergics (2 studies).

### Types of adverse medication effects

The scales measured psychotropic medication adverse effects (MEDS, Neurological Side Effect Scale (NSEC), Barkley’s Side Effect Rating Scale (SERS), UKU), more specific movement-related adverse medication effects (Akathisia Ratings of Movement Scale (ARMS), Dyskinesia Identification System Condensed User Scale (DISCUS)), cognitive ability (Hamburg Wechsler Intelligenztest für Erwachsene (HAWIE-R), Five Point Test, Regensburger Wortflüssigkeitstest (RWT), Rivermead Behavioural Memory Test (RBMT), Trail Making Test), stereotyped behaviour (Stereotyped Behaviour Scale), tics (Yale-Brown Global Tic Severity Scale) and obsessive–compulsive symptoms (Yale-Brown Obsessive–Compulsive Scale).

### Psychometric properties

One document examined the psychometric properties of two scales, MEDS and ARMS [32]. Both scales were found to be valid, i.e. both scales correctly indicated akathisia in those individuals with a diagnosis of akathisia and did not indicate akathisia in those without a diagnosis. Both scales were also found to be reliable, with high inter-rater agreement and internal consistency.

### Quality assessment

All studies included in this scoping review were accepted under the criteria for one of the MMAT categories and were of sufficient quality (Online Resource 5).

## Discussion

The accurate and timely detection of adverse effects of medication can significantly impact on quality of life and activities of daily living for people with ID [56]. This study examined the existing literature on medication adverse effect rating scales used in people with ID, the medication classes included in these scales, types of adverse medication effects identified and the available evidence on psychometric properties of these scales. Fifteen studies were deemed eligible for inclusion in the review and fourteen rating scales were identified which assessed the side effects of medications, primarily psychotropics, among people with ID.

### Availability of scales

There remains a paucity of research conducted in this field despite the widespread knowledge that people with ID are more susceptible to the detrimental adverse effects of medication due to unique abnormalities in neurological functioning and drug processing [57]. Of the fourteen rating scales identified in the review, only one was specifically developed for use in people with ID—the MEDS scale. Eight studies utilised the MEDS scale or subscale to identify adverse effects of medication. This scale has been described as the most reliable and most researched scale in participants with ID [34]. However, a substantial proportion of the sources which used the MEDS scale, and subsequently their associated results, were obtained through analysis of participants drawn from the same cohort by the same researchers.

### Scales investigated in children with ID

Four scales identified in this review were only investigated in participants aged under 18 years. These scales were the Stereotyped Behaviour Scale, Barkley’s SERS, Yale-Brown Global Tic Severity Scale and Yale-Brown Obsessive–Compulsive Scale.

The results obtained from the research of Correia Filho et al. suggest that observations from the two scales used in the study, UKU and Barkley’s SERS, correlate with the adverse medication effect profile observed from the use of risperidone and methylphenidate in the general population with average IQ [47]. The association of methylphenidate with insomnia, decreased appetite, weight loss and gastrointestinal adverse effects has been extensively documented in previous literature concerning the general population [58, 59]. Similarly, research has identified somnolence and weight gain as adverse effects of risperidone use in children with normal IQ [60]. These findings are akin to the results obtained from the sample of children and adolescents with moderate ID in this study. Although in this study the UKU and Barkley’s SERS rating scales were not directly adapted to children with ID, the correlation of results suggests that these scales could have utility for monitoring psychotropic adverse effects in this group. Correia Filho et al. suggest that children with ID presenting with only ADHD symptoms could be initiated on first line treatment with methylphenidate to minimise any unnecessary weight gain associated with risperidone. Conversely, children experiencing weight loss could benefit from initial treatment with risperidone to control symptoms [47]. However, the small sample size of the study limits the robustness of this recommendation.

Efforts have also been made to adapt existing rating scales to be directly applicable to children with ID. Ghuman et al. utilised the developmental disorder version of the Yale-Brown Obsessive–Compulsive Scale to assess the effects of methylphenidate for the treatment of ADHD symptoms in children with ID [50]. This version of the scale was modified to facilitate language difficulties by addressing compulsive behaviour only. Ghuman et al. also employed three additional scales to assess the safety of methylphenidate in the treatment of ADHD in preschool children with ID. The scales chosen reflected established adverse effects of methylphenidate that were hypothesised to affect people with ID. The utilisation of multiple scales enabled the authors to examine a wide range of adverse medication effects in the study sample. The effects of methylphenidate on physical, emotional and social functioning, including movement and stereotypic behaviour, were all considered, factors which could not be assessed collectively through a single scale. Overall, this approach established a greater understanding of the extent of methylphenidate adverse effects for preschool children with ID.

### Scales investigated in adults with ID

Seven scales identified in this review were exclusively investigated in participants aged over 18 years. These scales were the ARMS, HAWIE-R, Five-Point Test, MEDS, RWT, RBMT and Trail-Making Test.

Garcia et al. established that the MEDS and ARMS scales were effective in the assessment of symptoms of akathisia in adults with ID [49]. Interestingly, the two scales use different methods of data collection; the MEDS is an interview assessment, whereas the ARMS is an interactive observation assessment. When used in combination, the different methods of assessment allow for greater diagnostic clarity. Moreover, the two scales evaluate somewhat different symptoms, increasing coverage of the range of akathisia symptoms.

In assessing the adverse effects of TD due to the long-term use of atypical antipsychotics in people with ID, both the DISCUS and subscale of the MEDS scale (Central Nervous System-Parkinsonism/Dyskinesia) were utilised by Fodstad et al. The DISCUS scale has been previously shown to have an excellent convergent validity with the Central Nervous System-Parkinsonism/Dyskinesia subscale [48]. The Central Nervous System-Parkinsonism/Dyskinesia subscale was the primary scale utilised in this study. This is possibly due to the Central Nervous System-Parkinsonism/Dyskinesia subscale’s ability to distinguish TD from similarly presenting side-effects, whereas the DISCUS scale has been designed to measure dyskinesia only, rather than a range of side-effects that may be associated with psychotropic medications [35]. Furthermore, parkinsonism and dystonia are additional items that are only included in the MEDS subscale and not the DISCUS scale. These elements are important in assessing the extrapyramidal effects of psychotropic medications. Nonetheless, due to the significant correlation between both scales, both can be beneficial in assessing TD in people with ID.

### Scales identified in both children and adults with ID

Three scales identified in this review have been assessed in both adults and children with ID. These scales were the DISCUS, NSEC and UKU.

Hellings et al. employed a rating scale and a checklist, DISCUS and NSEC, to evaluate the adverse medication effects in people taking risperidone vs. placebo both in acute phase and long term treatment [52]. The use of both the scales and the checklist was effective in investigating the various adverse medication effects that were present in participants, with the DISCUS focusing on dyskinesia-related side effects and the NSEC focusing on neuroleptic side effects such as gastrointestinal upset, tremor and urinary incontinence. This allowed for greater insight into adverse effects caused by the medication in comparison to the placebo. The study design, an acute phase of 22 weeks and a 24-week follow up, allowed for the DISCUS and NSEC scales to monitor adverse effects of the drug over both a shorter and longer period of time.

Tveter et al. demonstrated the ability to adjust an established rating scale, making it more applicable to use for adults with ID. In this study, the revised UKU scale consisted of 35 items of the original 48-item scale that can be observed in people with ID [41]. Although it is a condensed scale, little information appears to be lost and adverse medication effects can be evaluated with increased accuracy. This has time and clinical implications in practice for monitoring medication-related adverse effects through observation in people with ID.

### Classes of medication

The scales identified in this review were predominantly used to assess adverse effects of psychotropic medication. A significant positive correlation was observed between the use of multiple psychotropic medications across different classes and an increased adverse medication effects profile.

The small, observational study by Matson et al. revealed a significant difference in MEDS severity ratings between individuals prescribed no psychotropics, those prescribed a selective serotonin reuptake inhibitor and those taking a selective serotonin reuptake inhibitor plus additional psychotropics [38]. The participants taking multiple psychotropic agents reported the highest adverse medication effects burden, followed by those exclusively taking a selective serotonin reuptake inhibitor.

These results were mirrored by a second study which emphasised that the more medications administered to people with ID, the greater the risk of untoward, largely irreversible adverse medication effects occurring [54]. This argument was supported by the results of the MEDS scale, which demonstrated that individuals prescribed two psychotropics scored significantly higher in the Central Nervous System-Behavioural/Akathisia subscale than individuals on a single agent. Furthermore, individuals prescribed several psychotropics had significantly higher sores on the Central Nervous System-Parkinsonism/Dyskinesia subscale than either the control or single psychotropic group.

Matson et al. investigated the use of the MEDS scale to assess adverse effects of antipsychotic medications in people with ID with comorbid TD and TD/akathisia [39]. Antipsychotic-related akathisia is well-established, and it is possible that the symptoms of TD and akathisia are heightened by antipsychotic medication [39, 40]. They concluded that people with TD/akathisia experienced an increased number of adverse medication effects than the groups without TD and with TD alone. The clinical significance of these findings has yet to be explored as further research is required with regards to how to manage and treat these adverse medication effects. However, it was emphasised that early detection is the best method for preventing such adverse medication effects.

### Scale robustness

Only one study conducted by Garcia et al. in 2006 [32] examined the psychometric validity of the MEDS and ARMS scales. The results from this study provided evidence of criterion validity. This allows the use of both scales in differentiating people with and without akathisia. However, concurrent validity was not demonstrated when both scales were correlated with each other. This could be explained by the fact that there is a variability in presented symptoms, particularly that akathisia symptoms vary in mild cases and over time. The lack of correlation between the scales suggests that both scales model different aspects of the akathisia construct. The most obvious difference is that the MEDS subscale combines both challenging behaviour and akathisia. This is supported by the higher Cronbach’s alpha in the MEDS than the ARMS subscale. One limitation of this paper is that it primarily focused on chronic akathisia and not acute drug-induced akathisia. Nevertheless, the authors concluded that as both scales demonstrated criterion validity but did not demonstrate concurrent validity, this would advise the recommendation for the multitrait-multimethod approach in assessing people with ID. However, multiple factors must be considered, including exposure to psychotropic medications, absence of non-drug causes, baseline behaviour and other movement disorders [32].

## Strengths and limitations

This review had many strengths. It is the only review to date, to our knowledge, in which information on all available rating scales used to measure medication adverse effects in people with ID is presented. A recent review by Copeland et al. examined measurement tools for adverse medication effect assessment in people with ID. However, this article focuses exclusively on rating scales designed to assess anti-epileptic adverse effects, as opposed to examining adverse medication effect scales in its entirety [61]. This review adopted a more comprehensive approach, with all medication-related adverse effects scales that are established as applicable, or potentially could be applicable to people with ID with appropriate adaptations, being examined. Inclusion and exclusion criteria were discussed and decided on by eight reviewers. Data extraction was completed by two reviewers and checked for accuracy and completeness by a third reviewer. Additionally, risk of bias was reduced in the screening process as two independent reviewers screened at each stage and disputes were resolved by a third reviewer. Few limits and filters were applied to each database search and all relevant articles from the past 20 years were retrieved. Articles before this time frame would not have much relevance in the current context of medication for people with ID as the landscape has evolved markedly in recent years. Forward and backward searching was performed on the references from included articles which created a more comprehensive representation of available scales. Critical appraisal was carried out using the MMAT. All studies included were deemed to meet the quality criteria outlined and had a low-medium risk of bias.

This review also had several limiting factors. While four major databases were searched for relevant articles, searching of other electronic databases may have resulted in a more comprehensive review. Reviewers were limited in the comparison and categorisation of studies due to the heterogeneity of the studies included. It was not possible to adopt a meta-analytic approach to combine the results of the studies due to variety in study design, outcome measures and outcomes assessed. Elements of PRISMA such as a quantitative assessment of internal biases were not performed as part of this study.

## Conclusions and future work

People with ID are often diagnosed with multiple comorbidities and are prescribed complex medication regimens to manage their conditions. It can be difficult for healthcare professionals to recognise adverse medication effects due to the difficulties in communication with this group. This review demonstrates an overall lack of suitable scales in assessing adverse effects of medications across domains in people with ID. The MEDS scale appears to be the most reliable and well researched scale in people with ID. However, when determining the robustness of this scale in comparison with the ARMS, concurrent validity was not demonstrated due to the variability of the symptoms present. Nevertheless, several studies reported the benefit of the use of multiple scales in assessing adverse effects of medications in people with ID. The utilisation of multiple scales provided a greater understanding and holistic approach to adverse medication effect monitoring when implemented. It is evident that more research needs to be carried out to determine the validity of these scales in assessing the adverse effects of medications in people with ID. In addition, the focus on psychotropic medication adverse effects across scales has resulted in a paucity of methods to determine adverse effects from other classes of medication. A scale which measures adverse effects across multiple medication classes would be valuable for use in this population.

## Supplementary Information

Below is the link to the electronic supplementary material.

Supplementary file1 (DOCX 39 KB)

Supplementary file2 (DOCX 566 KB)

Supplementary file3 (DOCX 26 KB)

Supplementary file4 (DOCX 31 KB)

Supplementary file5 (DOCX 31 KB)
