# Supplementary material for: Rating scales to measure adverse effects of medications in people with intellectual disability: a scoping review
Source: Eur J Clin Pharmacol. 2022 Aug 31;78(11):1711–25. doi: 10.1007/s00228-022-03375-2 (PMC9546988; doi:10.1007/s00228-022-03375-2)
Supplement: Supplementary file 1 — Supplementary file1 (DOCX 39 KB) [file 228_2022_3375_MOESM1_ESM.docx]

*Online Resource 1: Search strings and results retrieved*

| **Search Number** | **Search Terms** | **PsycINFO**  **No of Results** |
| --- | --- | --- |
| S1 | DE "Intellectual Development Disorder" OR DE "Anencephaly" OR DE "Crying Cat Syndrome" OR DE "Down's Syndrome" OR DE "Tay Sachs Disease" | 44,401 |
| S2 | DE "Side Effects (Drug)" OR DE "Drug Addiction" OR DE "Drug Allergies" OR DE "Drug Dependency" OR DE "Drug Sensitivity" OR DE "Side Effects (Treatment)" OR DE "Side Effects (Drug)" | 54,363 |
| S3 | TI ( DE "Intellectual Development Disorder" OR DE "Anencephaly" OR DE "Crying Cat Syndrome" OR DE "Down's Syndrome" OR DE "Tay Sachs Disease" ) OR AB ( DE "Intellectual Development Disorder" OR DE "Anencephaly" OR DE "Crying Cat Syndrome" OR DE "Down's Syndrome" OR DE "Tay Sachs Disease" ) | 44,401 |
| S4 | TI ( DE "Side Effects (Drug)" OR DE "Drug Addiction" OR DE "Drug Allergies" OR DE "Drug Dependency" OR DE "Drug Sensitivity" OR DE "Side Effects (Treatment)" OR DE "Side Effects (Drug)" ) OR AB ( DE "Side Effects (Drug)" OR DE "Drug Addiction" OR DE "Drug Allergies" OR DE "Drug Dependency" OR DE "Drug Sensitivity" OR DE "Side Effects (Treatment)" OR DE "Side Effects (Drug)" ) | 54,363 |
| S5 | S1 OR S3 | 44,401 |
| S6 | S2 OR S4 | 54,363 |
| S7 | scale* OR measure* OR indicator* OR tool* OR instrument* OR grad* OR rank* OR evalu* OR classific* | 2,042,747 |
| S8 | S5 AND S6 AND S7 | 129 |

| **Search Number** | **Search Terms** | **Medline**  **No of Results** |
| --- | --- | --- |
| S1 | (MH "Mentally Disabled Persons") OR (MH "Mentally Ill Persons") OR (MH "Intellectual Disability+") OR (MH "Mental Retardation, X-Linked+") OR (MH "Learning Disabilities+") | 124,171 |
| S2 | (MH "Drug-Related Side Effects and Adverse Reactions") | 31,520 |
| S3 | TI ( scale* OR measure* OR indicator* OR tool* OR instrument* OR grad* OR rank* OR evalu* OR classific* ) OR AB ( scale* OR measure* OR indicator* OR tool* OR instrument* OR grad* OR rank* OR evalu* OR classific* ) | 7,651,629 |
| S4 | "AB ( “Intellectual Disabil*” OR “Intellectually disabled” OR “mentally disabled” OR “mental disabil*” OR “learning disabil*” OR “Intellectual Development Disorder*” OR “Learning Disorder*” OR “Learning disabil*” OR “mental handicap*” OR “mentally handicapped” OR “mentally impaired” OR “mental impairment*” OR “intellectual impairment*” OR “developmental disabil*“ OR “mental defici*” OR “intellectual retard*” OR “mental retard*” OR “mentally retarded” OR “intellectually challenged” OR “intellectually deficient*” OR “intellectually handicapped” OR “intellectually impaired” OR “intellectually retarded” OR “mentally challenged” OR “mentally deficient” OR “mentally disabled person” ) OR TI ( “Intellectual Disabil*” OR “Intellectually disabled” OR “mentally disabled” OR “mental disabil*” OR “learning disabil*” OR “Intellectual Development Disorder*” OR “Learning Disorder*” OR “Learning disabil*” OR “mental handicap*” OR “mentally handicapped” OR “mentally impaired” OR “mental impairment*” OR “intellectual impairment*” OR “developmental disabil*“ OR “mental defici*” OR “intellectual retard*” OR “mental retard*” OR “mentally retarded” OR “intellectually challenged” OR “intellectually deficient*” OR “intellectually handicapped” OR “intellectually impaired” OR “intellectually retarded” OR “mentally challenged” OR “mentally deficient” OR “mentally disabled person” ) | 68,576 |
| S5 | "AB ( “drug-related side*” OR “drug related side*” OR “drug-related advers**” OR “drug related advers**” OR “chemical-related advers**” OR “chemical related advers**” OR “pharmaceutical-related advers**” OR “pharmaceutical related advers**” OR “Adverse Drug Event*” OR “Side Effects of Drug*” OR “Adverse Drug Reaction*” OR “drug adverse*” OR “drug-adverse*” ) OR TI ( “drug-related side*” OR “drug related side*” OR “drug-related advers**” OR “drug related advers**” OR “chemical-related advers**” OR “chemical related advers**” OR “pharmaceutical-related advers**” OR “pharmaceutical related advers**” OR “Adverse Drug Event*” OR “Side Effects of Drug*” OR “Adverse Drug Reaction*” OR “drug adverse*” OR “drug-adverse*” ) | 20,885 |
| S6 | S1 or S4 | 153,992 |
| S7 | S2 or S5 | 47,308 |
| S8 | S3 AND S6 AND S7 | 45 |

| **Search Number** | **Search Terms** | **Web of Science No of Results** |
| --- | --- | --- |
| S1 | TITLE: (“Intellectual Disabil*” OR “Intellectually disabled” OR “mentally disabled” OR “mental disabil*” OR “learning disabil*” OR “Intellectual Development Disorder*” OR “Learning Disorder*” OR “Learning disabil*” OR “mental handicap*” OR “mentally handicapped” OR “mentally impaired” OR “mental impairment*” OR “intellectual impairment*” OR “developmental disabil*“ OR “mental defici*” OR “intellectual retard*” OR “mental retard*” OR “mentally retarded” OR “intellectually challenged” OR “intellectually deficient*” OR “intellectually handicapped” OR “intellectually impaired” OR “intellectually retarded” OR “mentally challenged” OR “mentally deficient” OR “mentally disabled person ”) OR TOPIC: (“Intellectual Disabil*” OR “Intellectually disabled” OR “mentally disabled” OR “mental disabil*” OR “learning disabil*” OR “Intellectual Development Disorder*” OR “Learning Disorder*” OR “Learning disabil*” OR “mental handicap*” OR “mentally handicapped” OR “mentally impaired” OR “mental impairment*” OR “intellectual impairment*” OR “developmental disabil*“ OR “mental defici*” OR “intellectual retard*” OR “mental retard*” OR “mentally retarded” OR “intellectually challenged” OR “intellectually deficient*” OR “intellectually handicapped” OR “intellectually impaired” OR “intellectually retarded” OR “mentally challenged” OR “mentally deficient” OR “mentally disabled person ”) | 100,503 |
| S2 | TITLE: (“drug-related side*” OR “drug related side*” OR “drug-related advers**” OR “drug related advers**” OR “chemical-related advers**” OR “chemical related advers**” OR “pharmaceutical-related advers**” OR “pharmaceutical related advers**” OR “Adverse Drug Event*” OR “Side Effects of Drug*” OR “Adverse Drug Reaction*” OR “drug adverse*” OR “drug-adverse*”) OR TOPIC: (“drug-related side*” OR “drug related side*” OR “drug-related advers**” OR “drug related advers**” OR “chemical-related advers**” OR “chemical related advers**” OR “pharmaceutical-related advers**” OR “pharmaceutical related advers**” OR “Adverse Drug Event*” OR “Side Effects of Drug*” OR “Adverse Drug Reaction*” OR “drug adverse*” OR “drug-adverse*”) | 28,287 |
| S3 | TITLE: (scale* OR measure* OR indicator* OR tool* OR instrument* OR grad* OR rank* OR evalu* OR classific*) OR TOPIC: (scale* OR measure* OR indicator* OR tool* OR instrument* OR grad* OR rank* OR evalu* OR classific*) | 15,357,970 |
| S4 | S1 AND S2 AND S3 | 30 |

| **Search Number** | **Search Terms** | **OpenGrey**  **No of Results** |
| --- | --- | --- |
| S1 | TITLE: (scale* OR measure* OR indicator* OR tool* OR instrument* OR grad* OR rank* OR evalu* OR classific*) OR TOPIC: (scale* OR measure* OR indicator* OR tool* OR instrument* OR grad* OR rank* OR evalu* OR classific*) AND TITLE: (“drug-related side*” OR “drug related side*” OR “drug-related advers**” OR “drug related advers**” OR “chemical-related advers**” OR “chemical related advers**” OR “pharmaceutical-related advers**” OR “pharmaceutical related advers**” OR “Adverse Drug Event*” OR “Side Effects of Drug*” OR “Adverse Drug Reaction*” OR “drug adverse*” OR “drug-adverse*”) OR TOPIC: (“drug-related side*” OR “drug related side*” OR “drug-related advers**” OR “drug related advers**” OR “chemical-related advers**” OR “chemical related advers**” OR “pharmaceutical-related advers**” OR “pharmaceutical related advers**” OR “Adverse Drug Event*” OR “Side Effects of Drug*” OR “Adverse Drug Reaction*” OR “drug adverse*” OR “drug-adverse*”) AND TITLE: (“Intellectual Disabil*” OR “Intellectually disabled” OR “mentally disabled” OR “mental disabil*” OR “learning disabil*” OR “Intellectual Development Disorder*” OR “Learning Disorder*” OR “Learning disabil*” OR “mental handicap*” OR “mentally handicapped” OR “mentally impaired” OR “mental impairment*” OR “intellectual impairment*” OR “developmental disabil*“ OR “mental defici*” OR “intellectual retard*” OR “mental retard*” OR “mentally retarded” OR “intellectually challenged” OR “intellectually deficient*” OR “intellectually handicapped” OR “intellectually impaired” OR “intellectually retarded” OR “mentally challenged” OR “mentally deficient” OR “mentally disabled person ”) OR TOPIC: (“Intellectual Disabil*” OR “Intellectually disabled” OR “mentally disabled” OR “mental disabil*” OR “learning disabil*” OR “Intellectual Development Disorder*” OR “Learning Disorder*” OR “Learning disabil*” OR “mental handicap*” OR “mentally handicapped” OR “mentally impaired” OR “mental impairment*” OR “intellectual impairment*” OR “developmental disabil*“ OR “mental defici*” OR “intellectual retard*” OR “mental retard*” OR “mentally retarded” OR “intellectually challenged” OR “intellectually deficient*” OR “intellectually handicapped” OR “intellectually impaired” OR “intellectually retarded” OR “mentally challenged” OR “mentally deficient” OR “mentally disabled person ”) | 16 |
