# Supplementary material for: Rating scales to measure adverse effects of medications in people with intellectual disability: a scoping review
Source: Eur J Clin Pharmacol. 2022 Aug 31;78(11):1711–25. doi: 10.1007/s00228-022-03375-2 (PMC9546988; doi:10.1007/s00228-022-03375-2)
Supplement: Supplementary file 2 — Supplementary file2 (DOCX 566 KB) [file 228_2022_3375_MOESM2_ESM.docx]

*Online Resource 2 Chloropleth of study location by geographic region*


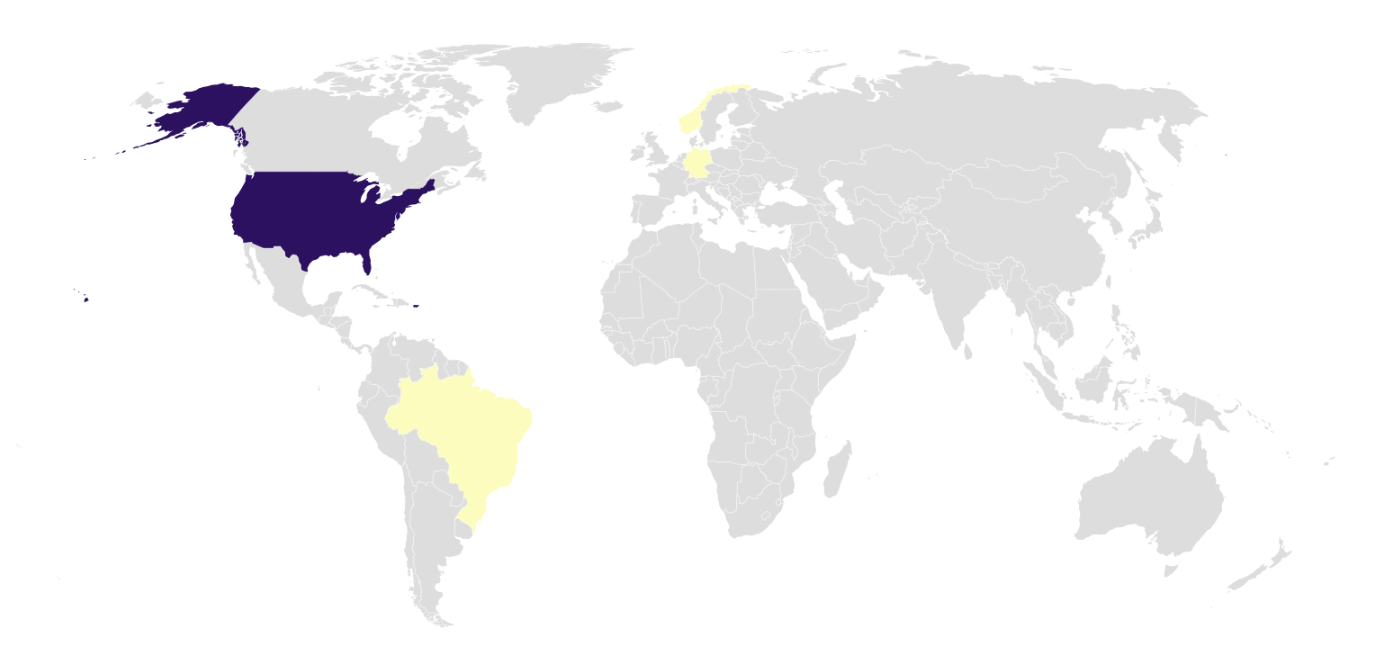


**USA**

**N=11**

Brazil

N=1

Norway

N=1

Germany

N=1

*Frequency Distribution*


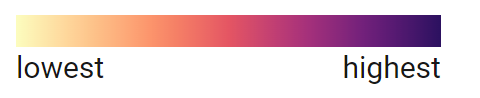

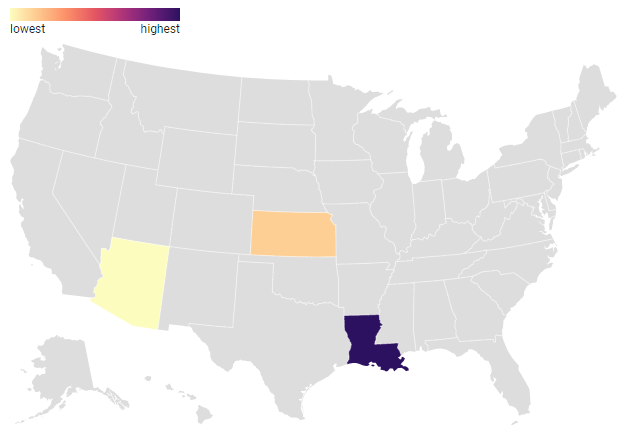


Louisiana

N=8

Kansas

N=2

Arizona

N=1
