# Supplementary material for: Rating scales to measure adverse effects of medications in people with intellectual disability: a scoping review
Source: Eur J Clin Pharmacol. 2022 Aug 31;78(11):1711–25. doi: 10.1007/s00228-022-03375-2 (PMC9546988; doi:10.1007/s00228-022-03375-2)
Supplement: Supplementary file 3 — Supplementary file3 (DOCX 26 KB) [file 228_2022_3375_MOESM3_ESM.docx]

*Online Resource 3 Summary of scales used in the studies discussed*

| Scale | Purpose | Description | Performance Measure |
| --- | --- | --- | --- |
| Akathisia Ratings of Movement Scale (ARMS) [1] | Movement-related adverse medication effects | Seven item interactive observation assessment of objective components of akathisia. Each item is scored on a Likert-type scale of 0 to 4, depending on severity. | Maximum score of 28.  A cut off score of 4 is used to define prevalence of akathisia. |
| Digit span forward and backward task from the Hamburg Wechsler Intelligenztest für Erwachsene (HAWIE-R) [2] | Cognitive ability | A sequence of numbers is read aloud to subjects and they are asked to repeat the same sequence of numbers in order (forward span) or in reverse order (backward span).  Forward span captures attention efficiency and capacity.  Backward span is an executive task particularly dependent on working memory. | Forwards and backwards performance can be reported either as sub scores (the number of correct items of each type) or as span scores (the maximum number of digits correctly produced forwards or backwards by the subjects). |
| Dyskinesia Identification System Condensed User Scale (DISCUS) [3] | Movement-related adverse medication effects | Fifteen dyskinesia items, rated from 0 to 4. | A scoring range of 0–60. A cut off score of 5 is defined as an at-risk score. |
| Five-Point Test [4] | Cognitive ability | Subjects are asked to produce as many different patterns as possible by connecting two, three, four or five points of a matrix within 3 minutes. | The number of correct patterns and the number of repetitions are used as a performance measure. |
| Matson Evaluation of Drug Side Effects Scale (MEDS) [5]* | Psychotropic medication adverse effects | Interview assessment of 90 items. Items are rated on a 3-point Likert-type scale with respect to severity and duration of specific symptoms that have occurred within the last 2 weeks. | Subscale scores are totalled to get composite scale score for overall severity. |
| Neurological Side Effect Scale (NSEC) [6] | Psychotropic medication adverse effects | A checklist for presence or absence of 29 potential adverse effects including gastrointestinal adverse effects, irritability, sedation, nasal congestion, fever, muscle stiffness, tremor, restlessness, and urinary incontinence and retention. | A scoring range of 0–29. |
| Regensburger Wortflüssigkeitstest (RWT) [7] | Cognitive ability | The subject is required to say as many words as possible from a special category (animals, food, names). One minute is assigned to each category. | The number of produced items is taken as the performance score. |
| Rivermead Behavioural Memory Test (RBMT) [8] | Cognitive ability | Immediately after the presentation of a story the subject is asked to repeat the story. After a 10-20-minute interval the subject is asked to recall the story again. | Score is built from the number of items the subject can recall from the story. Maximum of 21 items. |
| Stereotyped Behaviour Scale [9] | Stereotyped behaviour | Twenty-four item rating scale assessing frequency and severity of stereotyped behaviours. Each item is rated on a six-point frequency-of-occurrence scale and a four-point severity scale. | Higher scores reflect higher frequency and higher severity of the stereotyped behaviors. |
| The Barkley’s Side Effects Rating Scale (SERS) [10, 11] | Psychotropic medication adverse effects | A 4-point or 10-point scale. | Higher scores reflect higher severity of the side effect. |
| Trail Making Test adapted from the Delis Kaplan Executive Function System (D-KEFS) [12] | Cognitive ability | The subject is required to trace the numbers 1 to 16 and the letters A to P which are displayed in an irregular arrangement on a sheet of paper. The subject is required to alternate between numbers and letters in ascending order. | The time needed to complete the task is used as the performance score. |
| Udvalg for Kliniske Undersøgelser (UKU) Rating Scale [13] | Psychotropic medication adverse effects | Observational instrument, consisting of 48 items. Adverse medication effects are assessed in four domains: psychiatric, neurological, autonomic and other. The presence and severity of symptoms is considered and scored from 0 to 3. The probability of a correlation between adverse effects and medication is rated as ‘impossible’, possible, or ‘probable’. | Higher scores indicate presence and severity of adverse medication effects. |
| Yale-Brown Global Tic Severity Scale [14] | Tics | Tics are rated based on five grading criteria: number, frequency, intensity, complexity and interference. Five separate scores are obtained: Total motor tic score, total phonic tic score, total tic score, overall impairment rating and global severity score. | With increasing score, increasing severity of tics are observed. |
| Yale-Brown Obsessive-Compulsive Scale [15] | Obsessive-compulsive symptoms | A 10-item measure of obsession and compulsion severity. Items are rated over the previous week on a 5-point Likert-type scale ranging from 0 to 4. | Increasing score indicates increasing severity of obsessive-compulsive symptoms. |

**The MEDS scale is the only measure which has been developed specifically for use in a population with ID.*

**References:**

1. Bodfish JW, Newell KM, Sprague RL, Harper VN, Lewis MH (1997) Akathisia in adults with mental retardation: development of the Akathisia Ratings of Movement Scale (ARMS). Am J Ment Retard 101(4):413-23.

2. Tewes U. HAWIE-R. Hamburg-Wechsler Intelligenztest für Erwachsene. Revision 1991. Verlag Hans Huber.[RV]; 1991.

3. Sprague RL, Kalachnik JE, Shaw KM (1989) Psychometric properties of the Dyskinesia Identification System: Condensed User Scale (DISCUS). Mental retardation 27(3):141-8.

4. Regard M SE, Knapp P. Der Fuenf-Punkt Test. Zuerich: UniversitaetsSpital, Neurologische Klinik; 1982.

5. Matson JL, Mayville EA, Bielecki J, Barnes WH, Bamburg JW, Baglio CS (1998) Reliability of the Matson Evaluation of Drug Side Effects Scale (MEDS). Res Dev Disabil 19(6):501-6. <https://doi.org/10.1016/s0891-4222(98)00021-3>

6. Gualtieri C (1984) The Neuroleptic Side Effects Checklist. Unpublished scale available from the author.

7. Aschenbrenner S, Tucha O, Lange KW. Regensburger Wortflüssigkeits-Test. Hogrefe, Verlag für Psychologie; 2000.

8. Wilson B, Cockburn J, Baddeley A (1992) Der Rivermead behavioural memory test. Thames Valley Test Company

9. Rojahn J, Matlock ST, Tassé MJ (2000) The stereotyped behavior scale: Psychometric properties and norms. Research in Developmental Disabilities 21(6):437-54.

10. Barkley RA, McMurray MB, Edelbrock CS, Robbins K (1990) Side effects of methylphenidate in children with attention deficit hyperactivity disorder: a systemic, placebo-controlled evaluation. Pediatrics 86(2):184-92.

11. Barkley RA, G E (1998). Diagnostic interview, behavior rating scales, and the medical examination. Attention-deficit hyperactivity disorder: A handbook for diagnosis and treatment. Guilford Press, New York.

12. Delis DC, Kaplan E, Kramer JH. Delis-Kaplan executive function system. Psychological Corporation; 2001.

13. Lingjærde O, Ahlfors UG, Bech P, Dencker SJ, Elgen K (1987) The UKU side effect rating scale: A new comprehensive rating scale for psychotropic drugs and a cross-sectional study of side effects in neuroleptic-treated patients. Acta psychiatrica Scandinavica 76(s334):1-100. <https://doi.org/https://doi.org/10.1111/j.1600-0447.1987.tb10566.x>

14. Storch EA, Murphy TK, Geffken GR, Sajid M, Allen P, Roberti JW et al (2005) Reliability and validity of the Yale Global Tic Severity Scale. Psychological assessment 17(4):486.

15. Scahill L, McDougle CJ, Williams SK, Dimitropoulos A, Aman MG, McCracken JT et al (2006) Children's Yale-Brown Obsessive Compulsive Scale modified for pervasive developmental disorders. Journal of the American Academy of Child & Adolescent Psychiatry 45(9):1114-23.
