# Supplementary material for: Rating scales to measure adverse effects of medications in people with intellectual disability: a scoping review
Source: Eur J Clin Pharmacol. 2022 Aug 31;78(11):1711–25. doi: 10.1007/s00228-022-03375-2 (PMC9546988; doi:10.1007/s00228-022-03375-2)
Supplement: Supplementary file 4 — Supplementary file4 (DOCX 31 KB) [file 228_2022_3375_MOESM4_ESM.docx]

*Online Resource 4 Populations examined in included articles*

| Population | Scale Used | Study |
| --- | --- | --- |
| Children with ID | Stereotyped Behaviour Scale | Ghuman *et al*. 2009 [1] |
|  | Barkley’s SERS | Correia Filho *et al*. 2005 [2] |
|  |  | Ghuman *et al*. 2009 [1] |
|  | Yale-Brown Global Tic Severity Scale | Ghuman *et al*. 2009 [1] |
|  | Yale-Brown Obsessive-Compulsive Scale | Ghuman *et al*. 2009 [1] |
| Adults with ID | ARMS | Garcia. 2006 [3] |
|  |  | Garcia *et al*. 2008 [4] |
|  | HAWIE-R | Brandt *et al*. 2015 [5] |
|  | Five-Point Test | Brandt *et al*. 2015 [5] |
|  | MEDS | Fodstad *et al*. 2010 [6] |
|  |  | Garcia 2006 [3] |
|  |  | Garcia *et al*. 2008 [4] |
|  |  | Hess *et al*. 2010 [7] |
|  |  | Mahan *et al*. 2010 [8] |
|  |  | Matson *et al*. 2001 [9] |
|  |  | Matson *et al*. 2008 [10] |
|  |  | Matson *et al*. 2009 [11]  Matson *et al*. 2010 [12] |
|  | RWT | Brandt *et al*. 2015 [5] |
|  | RBMT | Brandt *et al*. 2015 [5] |
|  | Trail Making Test | Brandt *et al*. 2015 [5] |
| Both Populations | DISCUS | Matson *et al*. 2000 [12] |
|  |  | Hellings *et al*. 2006 [13] |
|  |  | Hellings *et al*. 2010 [14] |
|  | NSEC | Hellings *et al*. 2006 [13] |
|  |  | Hellings *et al*. 2010 [14] |
|  | UKU | Correia Filho *et al*. 2005 [2] |
|  |  | Tveter *et al*. 2014 [15] |

Abbreviations: ARMS, Akathisia Ratings of Movement Scale; DISCUS, Dyskinesia Identification System Condensed User Scale; D-KES, Delis Kaplan Executive Function System; HAWIE-R, Hamburg Wechsler Intelligenztest für Erwachsene; MEDS, Matson Evaluation of Drug Side-effects; NSEC, Neurological Side Effect Scale; SERS, Barkley’s Side Effects Rating Scale; RBMT, Rivermead Behavioural Memory Test; RWT, Regensburger Wortflüssigkeitstest; UKU, Udvalg for Kliniske Undersøgelser.

References

1. Ghuman JK, Aman MG, Lecavalier L, Riddle MA, Gelenberg A, Wright R et al (2009) Randomized, placebo-controlled, crossover study of methylphenidate for attention-deficit/hyperactivity disorder symptoms in preschoolers with developmental disorders. J Child Adolesc Psychopharmacol 19(4):329-39. <https://doi.org/10.1089/cap.2008.0137>

2. Correia Filho AG, Bodanese R, Silva TL, Alvares JP, Aman M, Rohde LA (2005) Comparison of Risperidone and Methylphenidate for Reducing ADHD Symptoms in Children and Adolescents With Moderate Mental Retardation. Journal of the American Academy of Child & Adolescent Psychiatry 44(8):748-55. <https://doi.org/10.1097/01.chi.0000166986.30592.67>

3. Garcia M (2006) Psychometric validity for the Matson Evaluation of drug side effects and the akathisia rating of movement scale. Dissertation, Louisiana State University

4. Garcia MJ, Matson JL (2008) Akathisia in adults with severe and profound intellectual disability: a psychometric study of the MEDS and ARMS. Journal of intellectual & developmental disability 33(2):171-6. <https://doi.org/10.1080/13668250802065190>

5. Brandt C, Lahr D, May TW (2015) Cognitive adverse events of topiramate in patients with epilepsy and intellectual disability. Epilepsy Behav 45:261-4. <https://doi.org/10.1016/j.yebeh.2014.12.043>

6. Fodstad JC, Bamburg JW, Matson JL, Mahan S, Hess JA, Neal D et al (2010) Tardive dyskinesia and intellectual disability: an examination of demographics and topography in adults with dual diagnosis and atypical antipsychotic use. Res Dev Disabil 31(3):750-9. <https://doi.org/10.1016/j.ridd.2010.01.017>

7. Hess J, Matson J, Neal D, Mahan S, Fodstad J, Bamburg J et al (2010) A Comparison of Psychotropic Drug Side Effect Profiles in Adults Diagnosed With Intellectual Disabilities and Autism Spectrum Disorders. Journal of Mental Health Research in Intellectual Disabilities 3(2):85-96. <https://doi.org/10.1080/19315861003690588>

8. Mahan S, Holloway J, Bamburg JW, Hess JA, Fodstad JC, Matson JL (2010) An Examination of Psychotropic Medication Side Effects: Does taking a greater number of psychotropic medications from different classes affect presentation of side effects in adults with ID? Res Dev Disabil 31(6):1561-9. <https://doi.org/https://doi.org/10.1016/j.ridd.2010.05.006>

9. Matson JL, Mayville EA, Bamburg JW, Scott Eckholdt C (2001) An analysis of side-effect profiles of anti-seizure medications in persons with intellectual disability using the Matson Evaluation of Drug Side Effects (MEDS). J Intellect Dev Disabil 26(4):283-95. <https://doi.org/10.1080/13668250120087308>

10. Matson JL, Rivet TT, Fodstad JC (2008) Matson Evaluation of Drug Side-effects (MEDS) Profiles in Adults with Intellectual Disability, Tardive Dyskinesia, and Akathisia. J Dev Phys Disabil 20(3):283-95. <https://doi.org/10.1007/s10882-007-9097-x>

11. Matson JL, Rivet TT, Fodstad JC (2009) Matson Evaluation of Drug Side-Effects (MEDS) Profiles of Selective Serotonin Reuptake Inhibitors (SSRI) in Adults with Intellectual Disability. J Dev Phys Disabil 21(1):57-68. <https://doi.org/10.1007/s10882-008-9125-5>

12. Matson JL, Rivet TT, Fodstad JC (2010) Atypical Antipsychotic Adjustments and Side-Effects over Time in Adults with Intellectual Disability, Tardive Dyskinesia, and Akathisia. J Dev Phys Disabil 22(5):447-61. <https://doi.org/10.1007/s10882-009-9179-z>

13. Hellings JA, Zarcone JR, Reese RM, Valdovinos MG, Marquis JG, Fleming KK et al (2006) A Crossover Study of Risperidone in Children, Adolescents and Adults with Mental Retardation. Journal of autism and developmental disorders 36(3):401-11. <https://doi.org/10.1007/s10803-006-0078-1>

14. Hellings JA, Cardona AM, Schroeder SR (2010) Long-Term Safety and Adverse Events of Risperidone in Children, Adolescents, and Adults With Pervasive Developmental Disorders. Journal of Mental Health Research in Intellectual Disabilities 3(3):132-44. <https://doi.org/10.1080/19315864.2010.494763>

15. Tveter A, Bakken T, Bramness J, Rossberg J (2014) Adjustment of the UKU Side Effect Rating Scale for adults with intellectual disabilities. A pilot study. Adv Ment Health Intellect Disabil 8:260-7. <https://doi.org/10.1108/AMHID-11-2013-0064>
