# Supplementary material for: Rating scales to measure adverse effects of medications in people with intellectual disability: a scoping review
Source: Eur J Clin Pharmacol. 2022 Aug 31;78(11):1711–25. doi: 10.1007/s00228-022-03375-2 (PMC9546988; doi:10.1007/s00228-022-03375-2)
Supplement: Supplementary file 5 — Supplementary file5 (DOCX 31 KB) [file 228_2022_3375_MOESM5_ESM.docx]

*Online Resource 5 Mixed Methods Appraisal Tool Quality Assessment of Included Studies*

| Author(s),  Year | All Study Types | | Quantitative Randomised Controlled Trials | | | | | Quantitative Non-Randomised Studies | | | | | Quality |
| --- | --- | --- | --- | --- | --- | --- | --- | --- | --- | --- | --- | --- | --- |
|  | Clear research questions | Collected data allow research questions to be addressed | Randomization appropriately performed | Groups comparable at baseline | Complete outcome data | Outcome assessors blinded to the intervention provided | Participants adhered to the assigned intervention | Participants representative of the target population | Measurements appropriate regarding both the outcome and intervention (or exposure) | Complete outcome data | Confounders accounted for in the design and analysis | Intervention administered (or exposure occurred) as intended |  |
| Brandt *et al.* 2015 [1] | Yes | Yes | - | - | - | - | - | Yes | Yes | Yes | Yes | Yes | Adequate |
| Correia Filho *et al.* 2005  [2] | Yes | Yes | Yes | Yes | Yes | Yes | Yes | - | - | - | - | - | Adequate |
| Fodstad *et al.* 2010 [3] | Yes | Yes | - | - | - | - | - | Yes | Yes | Yes | Yes | Yes | Adequate |
| Garcia 2006 [4]  Garcia *et al.* 2008 [5] | Yes | Yes | - | - | - | - | - | Yes | Yes | Yes | Yes | Yes | Adequate |
| Ghuman e*t al.* 2009 [6] | Yes | Yes | Yes | Yes | Yes | Yes | Yes | - | - | - | - | - | Adequate |
| Hellings *et al.* 2006  [7] | Yes | Yes | Unclear | Yes | Yes | Unclear | Yes | - | - | - | - | - | Adequate |
| Hellings *et al.* 2010 [8] | Yes | Yes | - | - | - | - | - | Yes | Yes | Yes | Yes | Yes | Adequate |
| Hess *et al.* 2010 [9] | Yes | Yes | - | - | - | - | - | Yes | Yes | Yes | Yes | Yes | Adequate |
| Mahan *et al.* 2010 [10] | Yes | Yes | - | - | - | - | - | Yes | Yes | Yes | Yes | Yes | Adequate |
| Matson *et al.* 2000 [11] | Yes | Yes | - | - | - | - | - | Yes | Yes | Yes | Yes | Yes | Adequate |
| Matson *et al.* 2001  [12] | Yes | Yes | - | - | - | - | - | Yes | Yes | Yes | Yes | Yes | Adequate |
| Matson *et al.* 2008  [13] | Yes | Yes | - | - | - | - | - | Yes | Yes | Yes | Yes | Yes | Adequate |
| Matson *et al.* 2009  [14] | Yes | Yes | - | - | - | - | - | Yes | Yes | Yes | Yes | Yes | Adequate |
| Matson *et al*. 2010  [15] | Yes | Yes | - | - | - | - | - | Yes | Yes | Yes | Yes | Yes | Adequate |
| Tveter *et al.* 2014 [16] | Yes | Yes | - | - | - | - | - | Yes | Yes | Yes | Yes | Yes | Adequate |

References:

1. Brandt C, Lahr D, May TW (2015) Cognitive adverse events of topiramate in patients with epilepsy and intellectual disability. Epilepsy Behav 45:261-4. <https://doi.org/10.1016/j.yebeh.2014.12.043>

2. Correia Filho AG, Bodanese R, Silva TL, Alvares JP, Aman M, Rohde LA (2005) Comparison of Risperidone and Methylphenidate for Reducing ADHD Symptoms in Children and Adolescents With Moderate Mental Retardation. Journal of the American Academy of Child & Adolescent Psychiatry 44(8):748-55. <https://doi.org/10.1097/01.chi.0000166986.30592.67>

3. Fodstad JC, Bamburg JW, Matson JL, Mahan S, Hess JA, Neal D et al (2010) Tardive dyskinesia and intellectual disability: an examination of demographics and topography in adults with dual diagnosis and atypical antipsychotic use. Res Dev Disabil 31(3):750-9. <https://doi.org/10.1016/j.ridd.2010.01.017>

4. Garcia M (2006) Psychometric validity for the Matson Evaluation of drug side effects and the akathisia rating of movement scale. Dissertation, Louisiana State University

5. Garcia MJ, Matson JL (2008) Akathisia in adults with severe and profound intellectual disability: a psychometric study of the MEDS and ARMS. Journal of intellectual & developmental disability 33(2):171-6. <https://doi.org/10.1080/13668250802065190>

6. Ghuman JK, Aman MG, Lecavalier L, Riddle MA, Gelenberg A, Wright R et al (2009) Randomized, placebo-controlled, crossover study of methylphenidate for attention-deficit/hyperactivity disorder symptoms in preschoolers with developmental disorders. J Child Adolesc Psychopharmacol 19(4):329-39. <https://doi.org/10.1089/cap.2008.0137>

7. Hellings JA, Zarcone JR, Reese RM, Valdovinos MG, Marquis JG, Fleming KK et al (2006) A Crossover Study of Risperidone in Children, Adolescents and Adults with Mental Retardation. Journal of autism and developmental disorders 36(3):401-11. <https://doi.org/10.1007/s10803-006-0078-1>

8. Hellings JA, Cardona AM, Schroeder SR (2010) Long-Term Safety and Adverse Events of Risperidone in Children, Adolescents, and Adults With Pervasive Developmental Disorders. Journal of Mental Health Research in Intellectual Disabilities 3(3):132-44. <https://doi.org/10.1080/19315864.2010.494763>

9. Hess J, Matson J, Neal D, Mahan S, Fodstad J, Bamburg J et al (2010) A Comparison of Psychotropic Drug Side Effect Profiles in Adults Diagnosed With Intellectual Disabilities and Autism Spectrum Disorders. Journal of Mental Health Research in Intellectual Disabilities 3(2):85-96. <https://doi.org/10.1080/19315861003690588>

10. Mahan S, Holloway J, Bamburg JW, Hess JA, Fodstad JC, Matson JL (2010) An Examination of Psychotropic Medication Side Effects: Does taking a greater number of psychotropic medications from different classes affect presentation of side effects in adults with ID? Res Dev Disabil 31(6):1561-9. <https://doi.org/https://doi.org/10.1016/j.ridd.2010.05.006>

11. Matson JL, Bamburg JW, Mayville EA, Logan JR (2000) Tardive Dyskinesia and Developmental Disabilities: An Examination of Demographics and Topography in Persons with Dual Diagnosis. The British Journal of Development Disabilities 46(91):119-30. <https://doi.org/10.1179/096979500799155711>

12. Matson JL, Mayville EA, Bamburg JW, Scott Eckholdt C (2001) An analysis of side-effect profiles of anti-seizure medications in persons with intellectual disability using the Matson Evaluation of Drug Side Effects (MEDS). J Intellect Dev Disabil 26(4):283-95. <https://doi.org/10.1080/13668250120087308>

13. Matson JL, Rivet TT, Fodstad JC (2008) Matson Evaluation of Drug Side-effects (MEDS) Profiles in Adults with Intellectual Disability, Tardive Dyskinesia, and Akathisia. J Dev Phys Disabil 20(3):283-95. <https://doi.org/10.1007/s10882-007-9097-x>

14. Matson JL, Rivet TT, Fodstad JC (2009) Matson Evaluation of Drug Side-Effects (MEDS) Profiles of Selective Serotonin Reuptake Inhibitors (SSRI) in Adults with Intellectual Disability. J Dev Phys Disabil 21(1):57-68. <https://doi.org/10.1007/s10882-008-9125-5>

15. Matson JL, Rivet TT, Fodstad JC (2010) Atypical Antipsychotic Adjustments and Side-Effects over Time in Adults with Intellectual Disability, Tardive Dyskinesia, and Akathisia. J Dev Phys Disabil 22(5):447-61. <https://doi.org/10.1007/s10882-009-9179-z>

16. Tveter A, Bakken T, Bramness J, Rossberg J (2014) Adjustment of the UKU Side Effect Rating Scale for adults with intellectual disabilities. A pilot study. Adv Ment Health Intellect Disabil 8:260-7. <https://doi.org/10.1108/AMHID-11-2013-0064>
